# Investigating spatiotemporal changes of the land-surface processes in Xinjiang using high-resolution CLM3.5 and CLDAS: Soil temperature

**DOI:** 10.1038/s41598-017-10665-8

**Published:** 2017-10-16

**Authors:** Xianyong Meng, Hao Wang, Yiping Wu, Aihua Long, Jianhua Wang, Chunxiang Shi, Xiaonan Ji

**Affiliations:** 1 State Key Laboratory of Simulation and Regulation of Water Cycle in River Basin & China Institute of Water Resources and Hydropower Research, Beijing, 100038 P. R. China; 2 Department of Earth & Environmental Science, Xi’an Jiaotong University, Xi’an, 710049 P. R. China; 3 National Meteorological Information Center, China Meteorological Administration, Beijing, 100081 P. R. China; 4 Xinjiang Institute of Ecology and Geography, Chinese Academy of Sciences, Urumqi, 830011 P. R. China

## Abstract

Soil temperature plays a key role in the land surface processes because this parameter affects a series of physical, chemical, and biological processes in the soil, such as water and heat fluxes. However, observation of soil temperature is quite limited, especially at the regional scale. Therefore, this study is to investigate the spatiotemporal features of soil temperature in Xinjiang, China, using the Community Land model 3.5 (CLM3.5) with the atmospheric near-surface forcing data of the China Meteorological Administration Land Data Assimilation System (CLDAS). We use the observed soil temperature data collected from 105 national automatic stations during 2009 through 2012 in the study area to verify the simulation capability. The comparison results indicate that the CLM3.5 with the CLDAS driving field could well simulate the spatiotemporal patterns of the soil temperature at hourly, daily, and monthly time scales and at three depths (5 cm, 20 cm, and 80 cm). We also produce a soil temperature database of the region that is continuous both in time and space with high resolution (about 6.25 km). Overall, this study could help understand the regional and vertical characteristics of the soil temperature and provide an important scientific basis for other land-surface processes.

## Introduction

Land surface accounts for one-third of Earth’s surface area. Extensive research has shown not only those land-surface conditions either directly or indirectly affect both climate and the atmosphere but also that anomalies in land-surface conditions constantly interact with both climate and the atmosphere^1–10^. It was Charney^11^ who, for the very first time, advanced the notion that changes in land surface will result in an abnormal change in the albedo, which in turn may further affect the surface radiation balance and eventually result in climate anomalies. Soil temperature and moisture are particularly important in the land-surface processes because they are key parameters that characterize the thermal properties and soil water content and can affect climate through their impacts on both surface energy and water budget^12^. Soil temperature could also impact the climate and the weather, especially on short-term weather processes^13^. In addition, soil temperature is an important agrometeorological element that affects a series of physical, chemical and biochemical processes in the soil. The most recent studies have shown that soil temperature can affect the short-term precipitation using the Eta model and the Weather Research and Forecasting model^14,15^. Hu and Feng^16^ found that the end-of-spring soil temperature is related to summer precipitation in some degree. The anomalies in the temperatures of shallow soil layers will affect short-term weather processes because the thermal anomalies in the surface layer’s soil have already been released to the atmosphere before being transferred downward to the deep layers. The anomalies in the soil of the deep layers can affect the regional climate process by gradually releasing energy to the shallow layers. Zhang *et al*.^17^ found a complex corresponding relationship between changes in the temperature of the atmosphere and that of the soil in Canada during the 20^th^ century. Wang *et al*.^18^ analyzed long-term trends of Eastern China’s soil temperature and moisture and strengthened the understanding of the mechanism and important characteristics of the land-atmosphere interaction in East China. However, measurement of soil temperature is deficient, and even if there are observations, they are spatially non-continuous and discrete.

Simulating the land-surface processes is a useful approach to studying the changes in the land-surface processes and their impacts, especially at the regional scale. Community Land Model (CLM3.5) is a good example, and many scientists pay much attention to the model verification and development, Bonan and Levis^19^ simulated the land surfaces of South America’s Amazon Plain and the eastern region of the US, respectively, using the CLM series dynamic vegetation model, the CLM3.0, coupled with the Community Atmospheric Model (CAM) (CLM3.0 and CAM3.0). They concluded that it was necessary to further improve the parameterization scheme of the CLM3.0 coupled with the CAM 3.0. CLM3.0 was updated to version 3.5 by modifying a series of land surface processes (e.g., C and N cycles, surface water, and snow accumulation and frozen soil), with a better performance especially in hydrological simulation^20^. Sakaguchi and Zeng^21^ proposed a flexible scheme about soil capacitance in CLM4.0, which was used to simulate the impacts of N cycle on the global carbon balance. CLM4.5 is the latest version of CLM, which has revised photosynthesis scheme, improved cold region hydrology and optional VIC-based hydrology, prognostic wetland distribution, a new snow cover fraction parameterization, a new lake model, new crop model features such as fertilization and grain fill, and multiple urban classes^22^, etc. However, we used CLM3.5 because applying CLDAS to drive CLM offline and its validation, while it is quite challenging to do so in CLM4.5, which deserves our future study.

Whitfield *et al*.^23^ have conducted a comparative study of simulation results for Florida using CLM and the Land Surface Process Model (LSPM), and found these two models were very good at simulating daily changes in soil temperature. Huang *et al*.^24^ stated that CLM3.0 could provide a very good simulation not only of various fluxes between the land and atmosphere but also of the characteristics of temporal and spatial distributions of soil temperature. Niu *et al*.^25^ conducted verification of three different types of land surface in East Asia (paddy field, sparse vegetation on plateaus and forest) using the CLM3.0. They found that surface temperatures in the sparse vegetation areas on the plateaus simulated using CLM3.0could fit the observed values relatively well and that CLM3.0could satisfactorily simulate the characteristics of the change in the soil temperature at different depth. Chen *et al*.^26^ investigated China’s soil temperature using CLM3.0driven by Princeton University’s global atmospheric forcing field data and found that the coupled model could provide a better simulation of the spatial distribution of soil.

From the above examples, we can see few scientists simulate the spatially continuous distribution of soil temperature at the regional scale. Generally, soil temperatures at a study point are from data collected at a nearby meteorological station, and this could cause large uncertainty especially in an area with complicated terrain^27^.

The Xinjiang Uyguy Autonomous Region, spans several latitudes and longitudes and has a very complicated topography. There is a lack of studies that used a land-surface model driven by high-precision atmospheric field data to simulate the continuous temporal and spatial distributions of the soil temperature in an area with a similar size. Therefore, the objective of this study is to 1) simulate soil temperature in the Xinjiang region using the CLM3.5 driven by the high-precision atmospheric driving field of the China Meteorological Administration Land Data Assimilation System (CLDAS), 2) evaluate the mode performance with observed data collected at 105 national automatic soil-temperature stations in the study area, and 3) investigate the characteristics of the spatiotemporal distributions of the soil temperatures at different depths.

## Results

### Monthly soil-temperature validation and features

**Figure 1:**
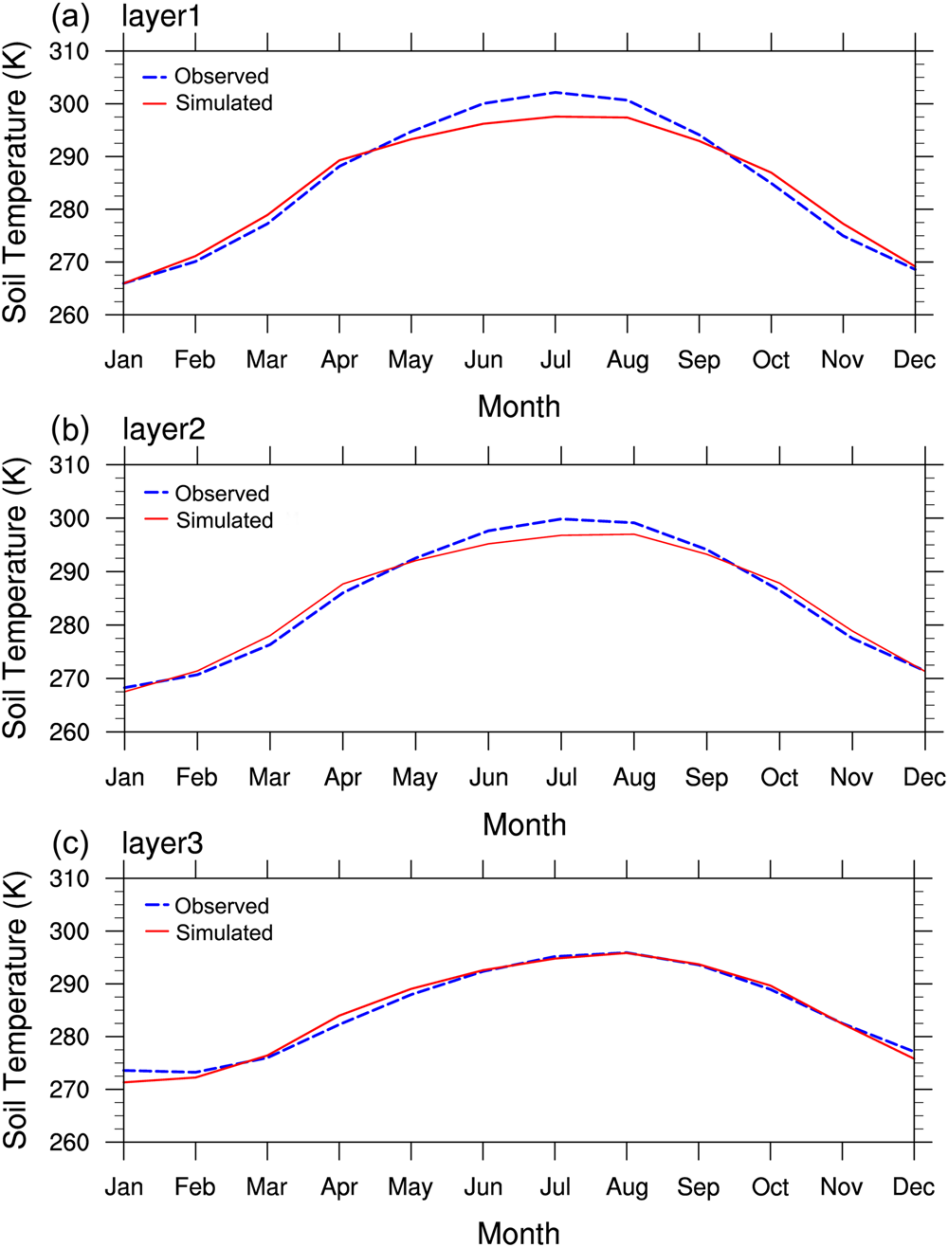
Simulation-observation curves of the monthly mean temperatures of the soil at 105 stations in Xinjiang at depths of (**a**) 5 cm, (**b**) 20 cm, and (**c**) 80 cm.

Figure 1 shows the simulated and observed monthly mean temperatures of the soil of three layers from 2009 to 2012. The results of the study show that the simulated temperatures of the soil are essentially consistent with the observed temperatures in terms of both the variation trend and the peak-to-valley value. The simulation results reflect the seasonal changes in the temperatures of the soil relatively well, indicating that the CLDAS-driven CLM3.5 can satisfactorily simulate the soil profile temperatures in Xinjiang. In addition, we find that the simulated soil temperatures in spring and autumn are the closest to their observations. From Fig. 1, the differences between the simulated and observed monthly mean temperatures of layer 1 soil in summer are the largest (approximately 8 K), followed by the differences between the simulated and observed monthly mean temperatures of layer 2 soil in summer (close to 5 K). The differences between the simulated and observed monthly mean temperatures of layer 3 soil in summer are the least. However, the simulated monthly mean temperatures of the soil of the two relatively shallow layers (layers 1 and 2) in January and December are relatively poor. In other words, the differences between the simulated and observed monthly mean temperatures of layer1 and layer2 soil in January and December are relatively large. We herein analyze the possible reasons for these differences. During the summer, the air temperature in the Xinjiang region changes dramatically, particularly in July, when the air temperature reaches its maximum value, which can be referred to as the extreme value of the air temperature. The surface temperature changes rapidly, but that change does not affect the soil of the deep layers. Therefore, the differences between the simulated and observed monthly mean temperatures of the soil of the shallow layers are larger than the difference between the simulated and observed monthly mean temperature of the soil of the deep layer. In winter, the cold transfers from the shallow layer to the deep layer (layer 3), which may be why the simulated monthly mean temperatures of the soil of the deep layer in winter are relatively poor.

**Figure 2:**
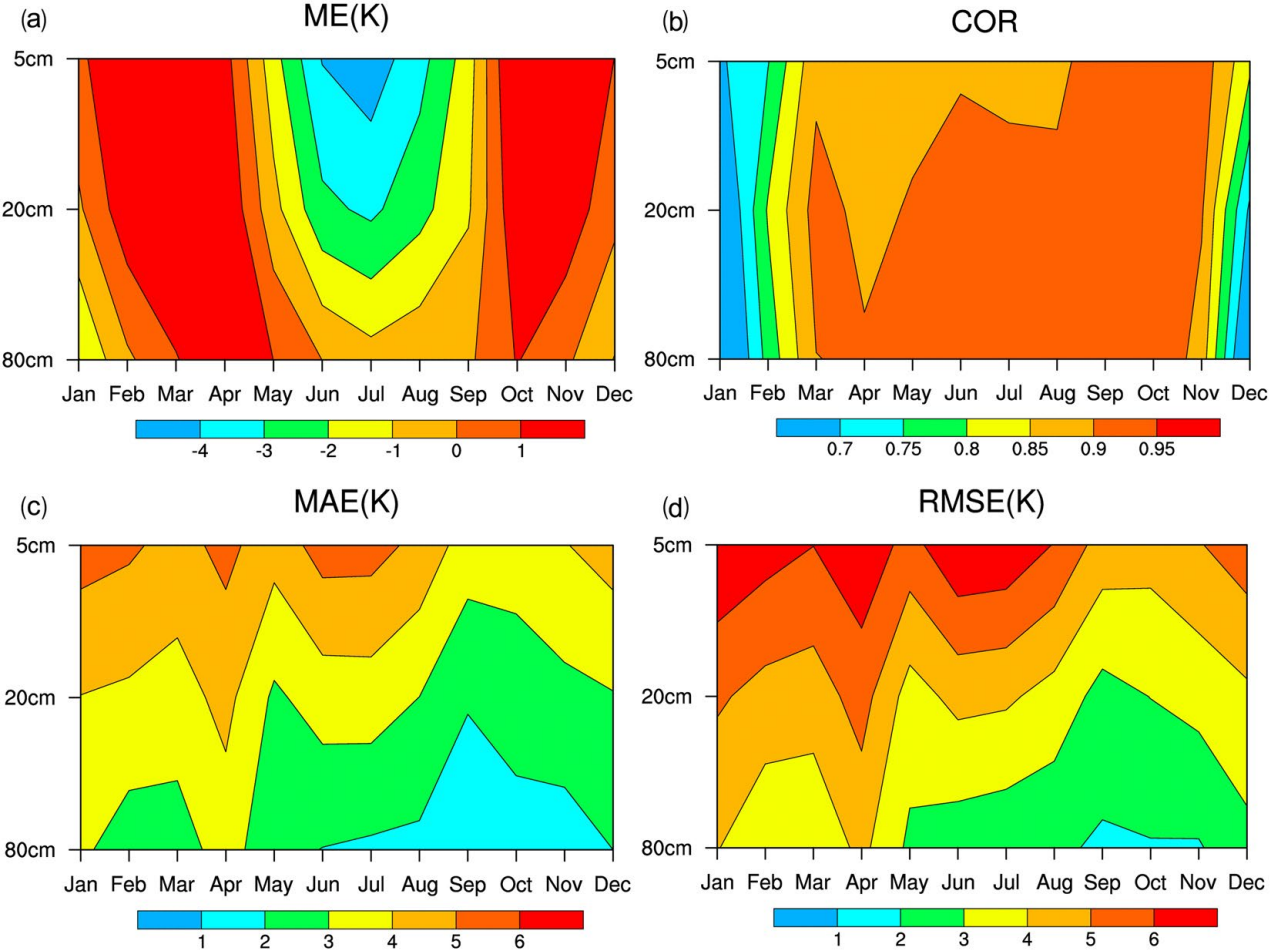
Statistical function graphs of the changes in the temperatures of the soil with season and depth. (**a**) ME, (**b**) Anomaly correlation, (**c**) MAE, and (**d**) RMSE.

Figure 2(a) shows the seasonal changes in the MEs of the soil temperatures at different depths. We can see that most of the simulated monthly mean temperatures of the soil of the three layers in summer (May to September) exhibit a negative deviation, but the simulated monthly mean temperatures of the soil of the three layers at all other times exhibit a positive deviation. Among the simulated monthly mean temperatures of layer 3 soil in all 12 months, only the simulated monthly mean temperature in January exhibits a positive deviation. Some of the simulated monthly mean temperatures of the soil of the deeper layer in summer also exhibit a positive deviation. From Fig. 2(b) that most of the anomaly correlation coefficients of the simulated monthly mean temperatures at three layers in all seasons are greater than 0.85, except for early spring and winter, indicating that CLDAS-driven CLM3.5 has a relatively good grasp on the pattern of the seasonal change in the soil temperature. In addition, we find that the anomaly correlation coefficients of layers 1 and 2 are less than those of layer 3. The anomaly correlation coefficients of layers 1 and 2 from May to September range from 0.85 to 0.9, whereas most of the anomaly correlation coefficients of layer 3 are 0.9. From the seasonal angle, the anomaly correlation coefficients of any of the three layers in early spring or late winter are relatively low (most of the anomaly correlation coefficients are below 0.7), which may be caused by the relatively low extreme value of the air temperature. Figure 2(c) and (d) show the seasonal changes in the MAEs and RMSEs, respectively, of the soil temperatures of different layers. We find that the spatial distribution pattern of the MAE is essentially similar to that of the RMSE. In addition, the simulation errors of layer 1 are greater than those of layer 2, and the errors of layer 3 are obviously the least. The errors of layers 1 and 2 in January, February, April, June, July and December are relatively large, whereas the errors of layer 3 are comparatively small.

### Daily soil-temperature validation and features

**Figure 3:**
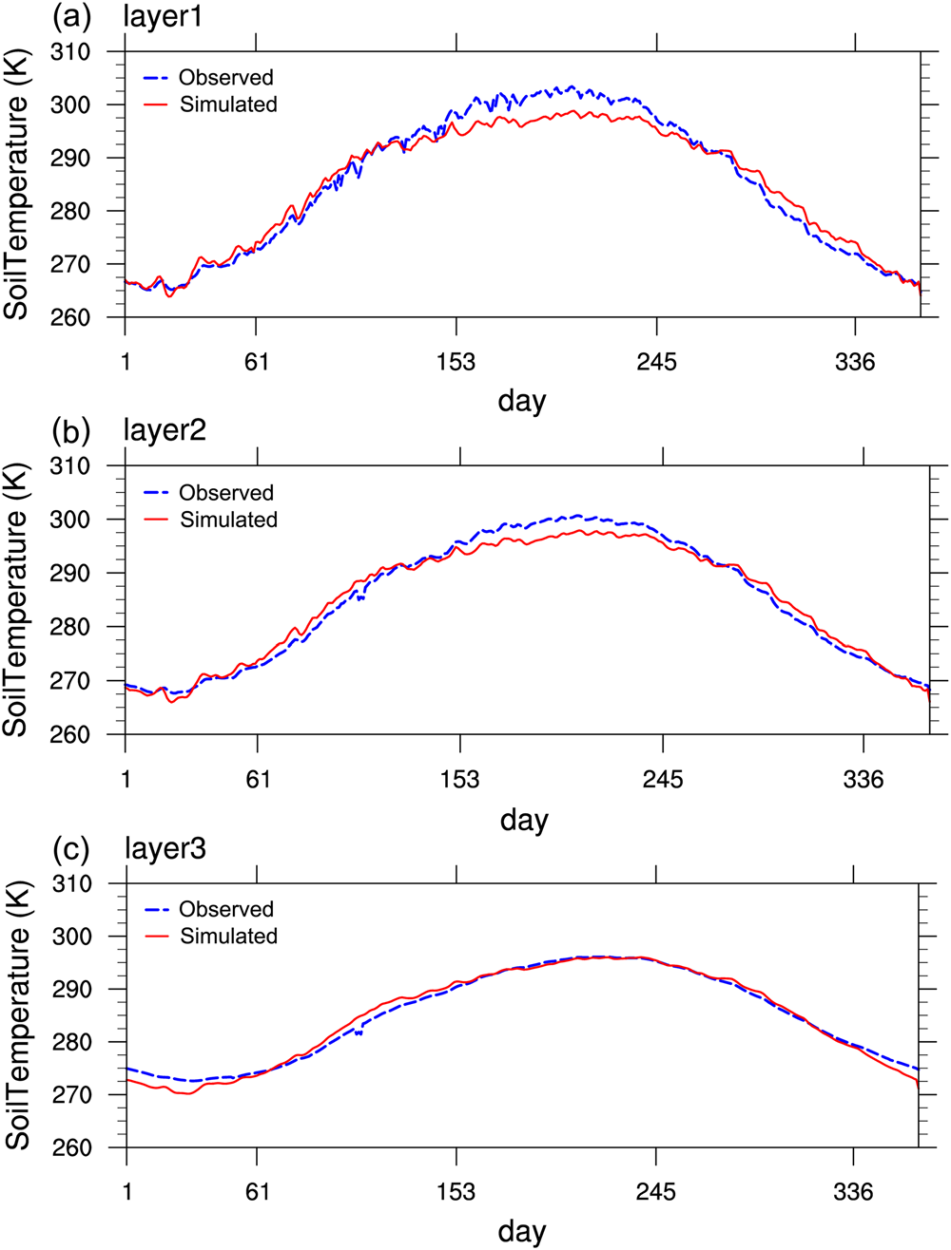
Simulation-observation curves of the daily mean temperatures of the soil at the 105 stations in Xinjiang (**a**) 5 cm; (**b**) 20 cm; (**c**) 80 cm.

Figure 3 shows the time-series plots of the 105-sample average daily soil temperature of three layers simulated using CLDAS-driven CLM3.5. The comparison between the simulations and the observations at the 105 national automatic-observation stations shows that the largest difference between is less than 5 K. From Fig. 3(a and b), the differences between the simulated and observed 105-sample average daily soil temperature of layer1 and layer2 in summer and autumn are the largest (around 5 K). The differences between the simulated and observed daily temperatures of layer 3 in January and December are greater than 2 K, whereas the differences in all other time periods are quite small. These results are consistent with the results of the changes in the monthly temperatures discussed in Section 4.1.

**Figure 4:**
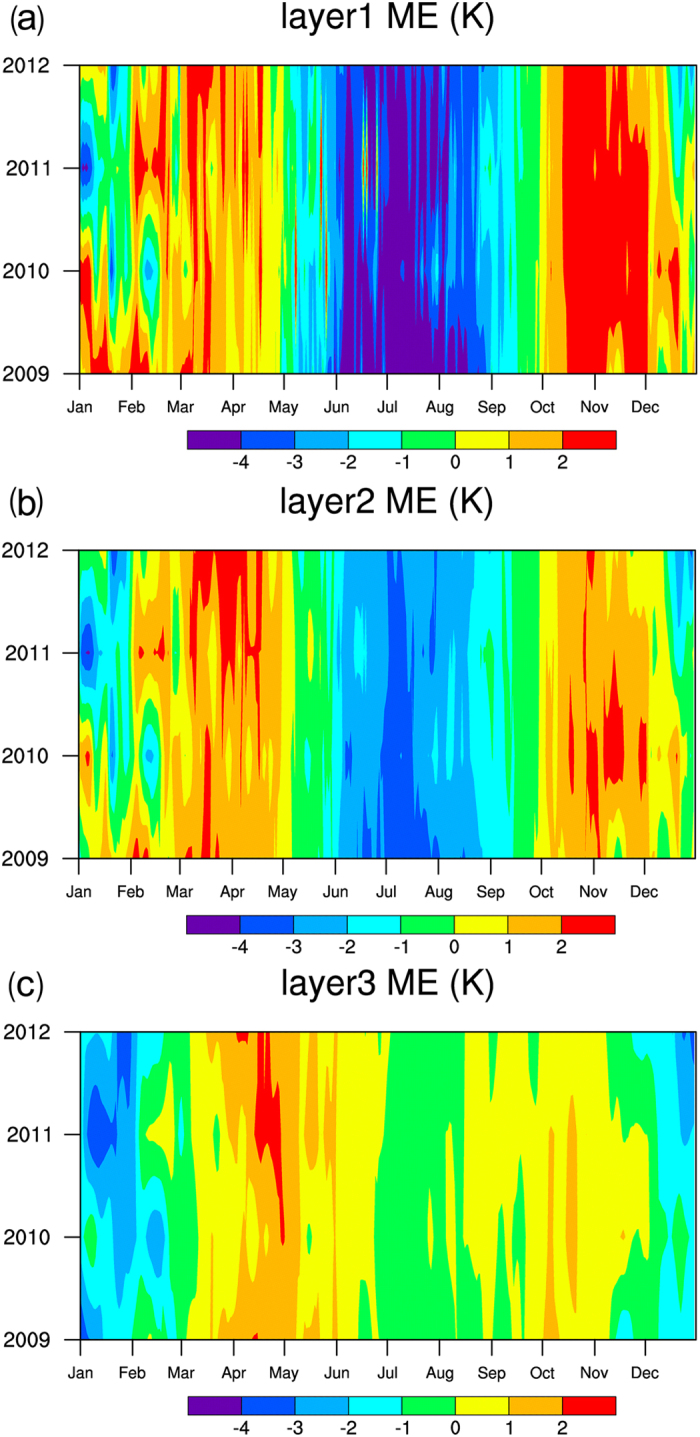
Seasonal changes in the mean deviations of the simulated daily mean temperatures from the observations in different years.

Figure 4(a–c) show the seasonal changes in the mean deviations of the daily mean temperatures in different years. It can be seen that the CLM3.5-simulated soil temperatures at the three layers in summer exhibit a deviation from the observed temperatures. The simulated summer temperatures of layer 1 and layer 2 exhibit a more significant deviation. The simulated temperatures of the surface layer from June to September of each year are approximately 2K–4K lower than the observed ones. The simulated soil temperatures of layer 2 from June to September of each year are approximately 1K–3K lower than the observed temperatures. In addition, the simulated temperatures of layer 1 and layer 2 from January to May and from October to November of each year also has a slight deviation (1K–2K). The simulated temperatures of layer 3 in January, February and December exhibit a relatively large deviation (1K–3K lower than the observed temperatures). The seasonal changes in the mean deviations of the simulated temperatures of layer 3 from the observed temperatures at all other times of the year mostly range from −1K to 1 K, suggesting that the seasonal fluctuation of the change in the soil temperature of layer 3 is less than that of layers 1 and 2.

### Hourly soil-temperature validation and features

**Figure 5:**
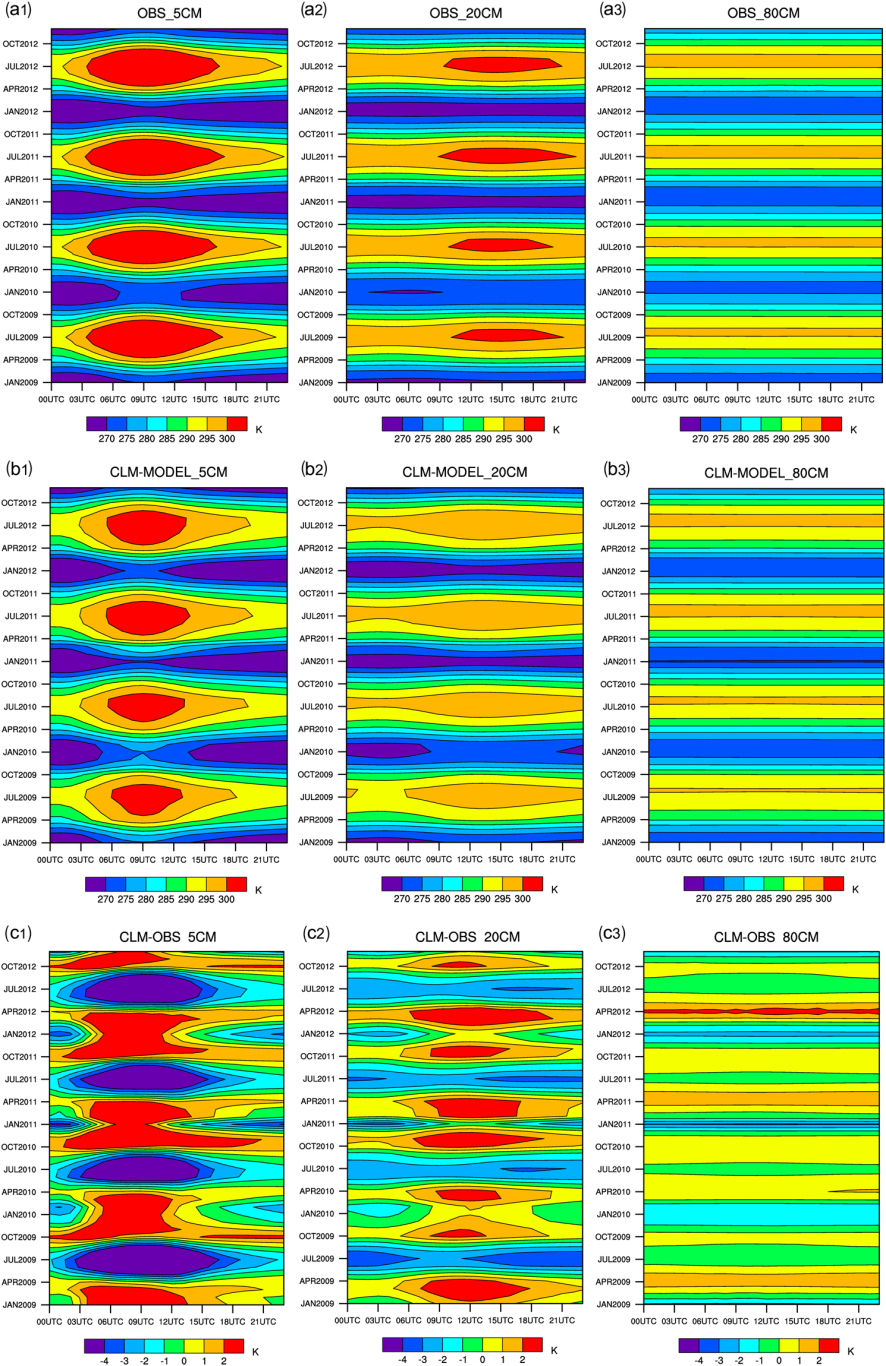
Annual and hourly changes in the observed (**a**) and simulated (**b**) soil temperatures at three depths and their differences (**c**).

Figure 5(a1–a3) shows the hourly soil temperatures at the three layers observed at the 105 stations in the Xinjiang region (a1: layer 1, a2: 20 cm, a3: 80 cm). The abscissa axis of each plot represents the coordinated universal time (UTC) and shows both the mean temperature distribution and the mean value of the change in the daily temperature over 24 hours at the 105 soil-temperature stations. The ordinate axis of each plot shows the change in the soil-temperature data from 2009 to 2012. We find that the inter-annual change in the temperature of layer 1 is larger than the inter-annual change in the temperature of layer 2 and the inter-annual change in the temperature of layer 3 on the daily scale. From February to November of each year, the temperature of layer 1 reaches its maximum value (above 30 °C) at 09UTC each day. In January and December of each year, the daily average temperature of layer 1 decreases to below 270 K. In addition, the largest difference between the daily maximum temperature and the daily minimum temperature of layer 1 exceeds 30 K in all time periods except for January and December. The daily change in the temperature of layer 2 is relatively slow and occurs at a later time. The daily maximum temperature of layer 2 soil (above 30 °C) occurs at 15UTC, indicating that there is a delay in the transfer of the soil temperature from a shallow layer to a deep layer. Furthermore, the temperature of layer 2 was lower than zero only in early January 2009, at the end of December 2010, in early January 2011, at the end of December 2011 and in January 2012.

Figure 5(a1–a3) also shows that there is essentially no change in the temperature of layer3 soil on the daily scale (i.e., over each 24 h day). The daily temperatures of layer 3 are between 290 K and 300 K from June to September. The highest daily temperatures of layer 3 are primarily concentrated in July (295K–300K), whereas the lowest daily temperatures of layer 3 are primarily concentrated in January. The early springs of 2011 and 2012 were relatively cold, resulting in daily temperatures of layer 3 in January and February being between 270 K and 275 K. The differences between the highest and lowest daily temperatures of layer 3 are between 15 K and 25 K, showing a gentler variation than layers 1 and 2. In addition, the energy between the land and atmosphere has a larger impact on the daily temperature of the surface layer than that on the deep layer.

In terms of the seasonal change in the soil temperature, the temperatures at all three layers of the soil reach their maximum values in summer and their minimum values in winter, suggesting clear seasonal changes.

Fig. 5(b1–b3) shows the distributions of the soil temperatures at the three layers ((b1), (b2) and (b3)) simulated using the CLDAS-driven CLM3.5. The simulated temperatures are interpolated to the matching latitudes and longitudes and depths of the 105 national automatic stations for statistical analysis. It is found that both the inter-annual change and the hourly change of each day in the soil temperature of each layer simulated using CLDAS-driven CLM3.5 fit the observed results very well, except for the simulated maximum temperature of layer 2 (15UTC). Overall, we could say the simulations of soil temperature at three soil depths by CLM3.5 in the Xinjiang region is acceptable.

Figure 5(c1–c3) shows a statistical analysis of the differences of the soil temperatures at the three layers between simulation and observation at the national automatic-soil temperature stations. Figure 5 tells that the simulation of soil temperatures is generally consistent with the observed ones. From the interpolation graph of the observed and simulated soil temperatures of layer1, we can see that the simulated temperatures of layer 1 between 03UTC and 21UTC from January to April and from September to November of each year are higher than their respective observed temperatures (0K–2K), whereas the simulated temperatures of layer 1 soil between 21UTC and 00UTC in the same time periods are lower than the observed temperatures (−1K–0K). In addition, the simulated temperatures of layer 1 throughout the day from May to August of each year are lower than the observed temperatures. The simulated maximum temperature of layer 1 occurs at 09UTC and is approximately 4 K lower than the observed maximum temperature. The reason for the relatively poor simulated values may be because the change in temperature in the Xinjiang region is the largest between 03UTC and 14UTC, and extreme values also occur in this time period. It is found that the differences between the simulated and observed temperatures of layer-1 soil are between −1 K and 1 K in all other non-extreme value time periods. The simulation results of the temperatures in non-extreme value time periods are ideal. The differences between the simulated and observed temperatures of layer 2 from January to April and from September to November of each year are mostly between −1 K and 1 K. Simulated temperatures of layer 2 at 12UTC reaches the maximum value, which is consistent with the information shown in Fig. 5(a2). We also find that the characteristics of the daily change in the soil temperature of layer 3 are not prominent. The simulated temperatures of layer 3 in late spring, summer and early autumn are 0–1 K higher than observations. The simulated temperatures of layer 3 in all other seasons are lower than the respective observed temperatures, and in winter the difference reach the largest (around 1–4 K) in winter.

## Discussion

The simulation of soil temperature is the key component of a land-surface model. The accuracy of the calculation of soil temperature using a land-surface model directly affects the material and energy exchanges between the land and the atmosphere in the model, which in turn affects the accuracy of the numerical model. This study uses the CLDAS data developed by the National Meteorological Information Center of China to force CLM3.5 to simulate the soil temperature in the Xinjiang region. This study describes both data integration and the core assimilation algorithm used in the preparation process of the CLDAS in details. This study validates and analyzes the soil temperatures at 105 national automatic stations in the Xinjiang region. The validation of soil temperature simulation using 105 national automatic stations indicated that CLM3.5 driven by CLDAS can simulate the changes of the soil temperatures at multiple layers in the Xinjiang region quite well, with the correlation coefficients being greater than 0.85. The deviations of the simulated values from the observed values are relatively small (no more than 2K–4K). The errors in the simulated soil temperatures of the shallow layers are relatively large, whereas they are relatively small for deep layers. Therefore, future studies should focus on further adjusting the parameters related to soil heat transfer and the parameterization schemes for other processes in the CLM to reduce the uncertainty of the model and obtain the optimal simulation results.

We admitted that there are still some drawbacks or limitations in our study. (1) From Fig. 2(a) and (b), we can see soil temperature in late winter and early spring was underestimated. Further, we also found some spikes (relatively high/low values) in the measurement data in this period, and this might be caused by the physical malfunction of probes during the melting period. The model did not perform well either in capturing this phenomenon. Actually, soil temperature variation involves water and energy fluxes and air temperature changes. The poor performance of soil temperature simulation from the late winter through the early spring due to the snow cover and air temperature were reported previously^28–31^. This complicated interaction complicated and abrupt change of soil temperature and poor model behavior deserves further investigation in future studies. (2) The soil temperature for the 105 stations were measured at a series depth of 5 cm, 10 cm, 15 cm, 20 cm, 40 cm, 80 cm, 160 cm, and 320 cm. The simulated soil temperature were for 10 soil layers with depths of 0.007 m, 0.0279 m, 0.0623 m, 0.1188 m, 0.212 m, 0.366 m, 0.619 m, 1.038 m, 1.727 m, 2.846 m. Our study was designed to take simulations from depths 0.0623 m, 0.366 m, and 1.038 m over the Xinjiang region for comparison with measurements from depths of 5 cm, 20 cm, and 80 cm. We understand this slight mismatch in depths could result in some biases including overestimation and underestimation, but it is unavoidable due to the model setting.

Application of numerical models usually involves input parameters uncertainty and its effects on model output, and this topic is quite attractive but challenging especially for large-scale studies. Many studies indicate that using an envelope of models could help assess the uncertainties and might reduce the systematic biases. Therefore, we are considering to investigate this issue in our future studies (1) using multiple models including CLM, Noah Land Surface Model, and/or the Common Land Model (CoLM) together with post statistical approaches such as Bayesian Model Averaging, and (2) using different climate and land-use scenarios to analyze the variabilities/uncertainties of model output^32^.

In spite of the above limitations and there was a national assessment of soil temperature of China using CLM3.0 with coarse resolutions (1° and 3-hour)^26^. In contrast, our model simulations can be more informative and useful because it investigates the spatial-temporal patterns of soil profile (at three depths) temperature in Xinjiang using CLM3.5 with high spatial (1/16°) and temporal (hourly) resolutions. The high-resolution input and output can help evaluate the spatial-temporal features of the land surface processes especially in local environment.

## Methods

### Study area

**Figure 6:**
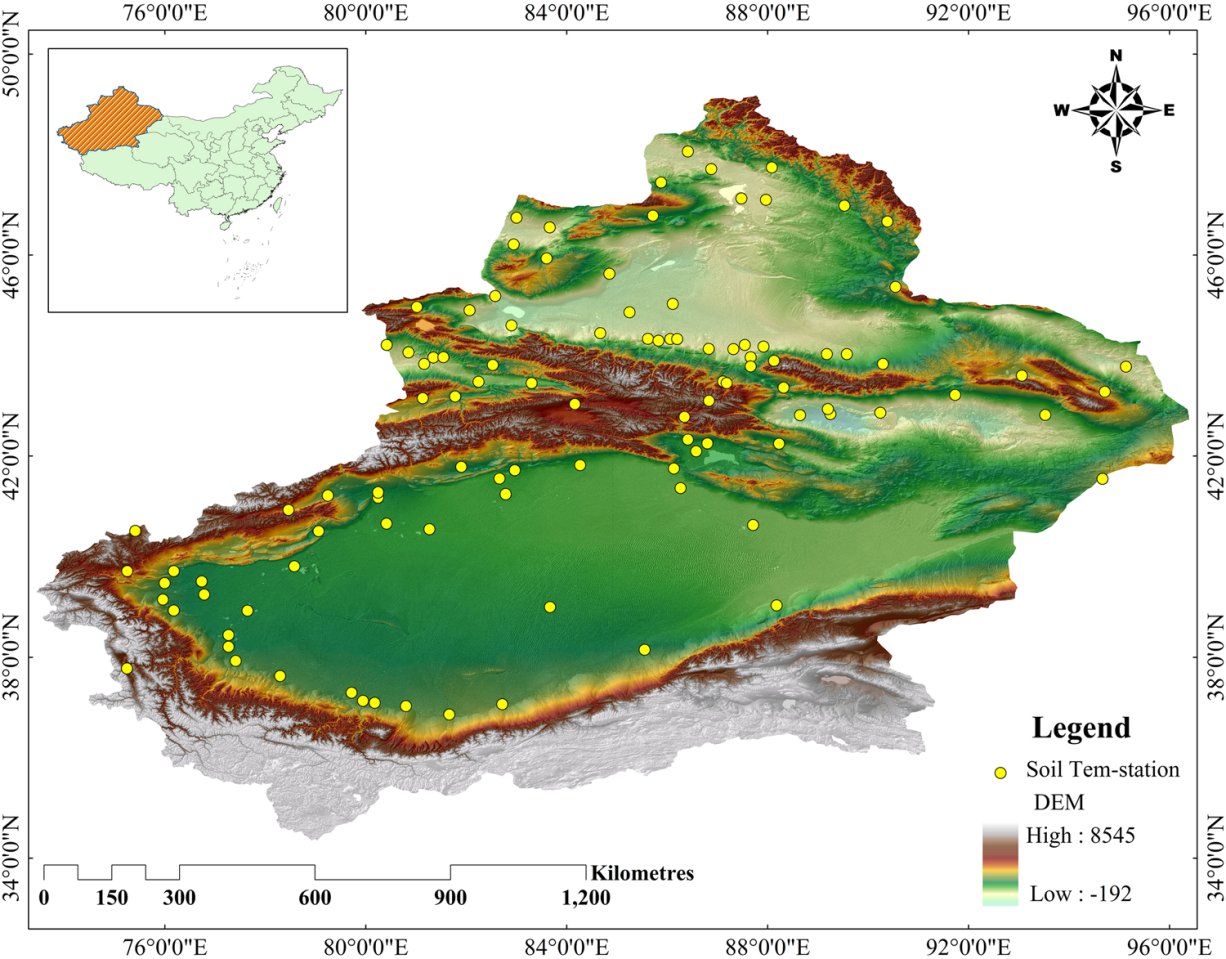
Location of the Xinjiang region and 105 automatic soil temperature observation stations. The map is generated with ArcMap Version 10.1 (http://www.esri.com/en/arcgis/arcgis-for-desktop/).

As shown in Fig. 6, Xinjiang Uyghur Autonomous Region is located in the hinterland of the Eurasian continent between 73°40′E and 96°18′E and between 34°25′N and 48°10′N. With a total area of 1.6649 × 10^6^ km^2^, Xinjiang accounts for nearly one-sixth of China’s total area and is the largest among the provincial-level administrative regions in China. With the closest coastline 2,648 km (straight-line distance) away, the Gurbantünggüt Desert in Xinjiang (46°16.8′N, 86°40.2′E) is the remotest point of land from any sea. There are alternating mountain ranges and basins across Xinjiang–basins surrounded by high mountains and high mountains surrounded by basins. The Altai Mountains are located in the north of Xinjiang, and the Kunlun Mountain system is located in the south of Xinjiang. The Tianshan Mountains span the center of Xinjiang and divide Xinjiang into southern and northern halves. The Tarim Basin is situated in the southern half, and the Dzungarian Basin is located in the northern half. Xinjiang’s unique landform-two basins sandwiched among three mountains, resulting in complicated underlying surface conditions and a huge spatial-temporal difference in climate distribution. Unfortunately, there are a deficient number of meteorological stations in the Xinjiang region, and literature review showed that most scientists just used reanalyzed data and/or some single-point data to study this region. Therefore, the existing studies on the land-surface processes in the Xinjiang region may be inaccurate or unsystematic.

### Models

The CLM developed by the US National Center for Atmospheric Research (NCAR) is used as the land-surface model in this study. This model was developed based on extensive, painstaking research on the numerical simulation of climate, vegetation ecology and watershed hydrology^33^. Possessing the advantages of existing land-surface models worldwide CLM is one of the most reliable land-surface process models in the world. CLM is the land-surface model component of the Community Earth System Model developed by the US NCAR^28^, and was primarily developed to couple with the earth-system model, the CLM can be operated offline (i.e., independently). When the CLM is operated offline, users need to provide the atmospheric forcing data, such as solar radiation, temperature, pressure, near-surface wind speed, specific humidity and precipitation rate. The CLM’s physical processes includes the heat and water exchange between the land and atmosphere, the dynamic growth processes of vegetation, the thermodynamic processes of soil and the hydrologic processes. These processes are primarily expressed using parameterization schemes.

The CLM series adopted the sub-grid variability of each model—each grid contains multiple types of land unit, such as glacier, lake, wetland, city and plant. The plant unit can also be divided into multiple plant function types (PFTs, i.e., tiles) (Fig. 6), and energy and water conservation must be maintained on each tile, which has its own diagnostic variable. Each tile in a lattice point obtains meant-state atmospheric forcing from the lattice point of the atmosphere corresponding to that lattice point. The tiles in a lattice point contribute their water and heat fluxes to the lattice point based on the proportion of the area. There is no direct interaction among the tiles in each lattice point. The CLM is divided into one plant layer, 10 soil layers of different thicknesses and a maximum of five snow layers (the number of snow layers is determined by the thickness of the snow) in the vertical direction. This layered structure is beneficial to obtain relatively high-resolution daily and seasonal changes in each layer’s temperature.

CLM3.5 was released by the US NCAR in 2007, and its modifications to CLM3.0consist of an improved hydrologic process and the introduction of new surface datasets^34–36^. The introduction of new surface datasets simultaneously improves the description of the land surface and the simulation of the surface albedo, surface temperature and precipitation. Niu *et al*.^35^ introduced a new frozen soil scheme. Oleson *et al*.^20^ and Lawrence *et al*.^34^ provided a detailed description of the physical processes and performance of CLM3.5.

CLM3.5 calculates soil temperatures using soil heat transfer equations:

The first law of heat transfer can be expressed as follows:

1 $$F=-\lambda {\rm{\Delta }}T$$

where $F$ represents the total amount of heat that transfers through a unit cross section area per unit of time ($W{m}^{2}$); $\lambda$ represents thermal conductivity ($W{m}^{-1}{K}^{-1}$); and ${\rm{\Delta }}T$ represents the spatial gradient of temperature ($K{m}^{-1}$). The one-dimensional (1D) form of the first law of heat transfer is expressed as follows:

2 $${F}_{z}=-\lambda \frac{\partial T}{\partial z}$$

where $z$ represents the vertical direction (m) and is positive downward; and ${F}_{z}$ is positive upward. To illustrate unstable or transient conditions, the principle of conservation of energy is called using the following continuity equation:

3 $$c\frac{\partial T}{\partial t}=-\frac{\partial {F}_{z}}{\partial z}$$

where $c$ represents the heat capacity of the snow/soil of the equivalent body ($J{m}^{-3}{K}^{-1}$); and $t$ represents time (s).

By simultaneously solving equations (2) and (3), the 1D form of the second law of heat transfer can be obtained:

4 $$c\frac{\partial T}{\partial t}=\frac{\partial }{\partial z}[\lambda \frac{\partial T}{\partial z}]$$

The soil and snow temperatures can be calculated by numerically solving the above equations.

Five additional snow cover layers are added on top of the 10 layers of soil columns. In addition, boundary condition h, as heat flux, enters from the above atmosphere layer to the snow/soil layer on the surface. The bottom layer of the soil volume has zero heat flux. Initially, there is no change in the period when calculating the temperature profile, and then the adjustments are made during the process in which the period changes.

### Experiments

In this study, the areas of lakes and wetlands are from the perennial freshwater lake and marshland data released by Cogley^37^. The soil colors are determined based on the results of the study by Zeng *et al*.^38^ and Dickinson *et al*.^39^. We use the geosphere biosphere program (IGBP) data of soil sand and clay content with 4931 image elements for each soil layer^40^ to generate a set of soil database that changes with the depth and soil texture^41,42^. PFTs and their contents along with the leaf area index are obtained based on the retrieval of the 1 km satellite data performed by Bonan *et al*.^42^. The glacier data are obtained from the IGBP data and the 1 km land surface cover database of the global information system (IGBP DISCover)^43^. The data of the stem area index and the heights of the upper and lower layers of each canopy are from Bonan *et al*.^42^.

**Figure 7:**
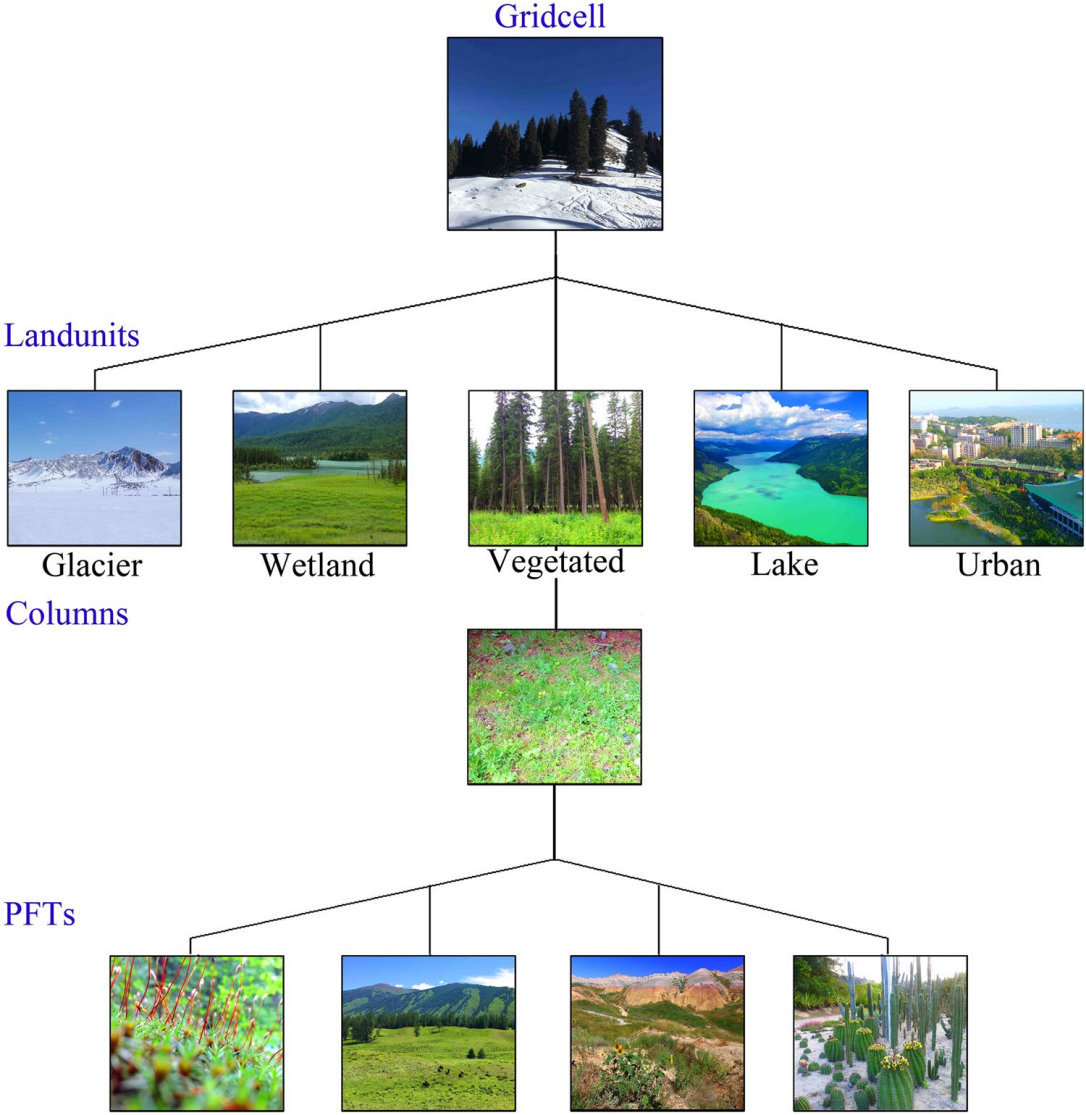
The sub grid distribution in CLM3.5 model.

Table 1 lists the land-surface data needed for each land-surface grid, including the proportions of glaciers, lakes and cities in each grid (the rest of the grid is occupied by plants). In addition, Fig. 7 lists the data of four PFTs with the highest contents, including the cover rates of each PFT, the leaf area index and stem area index of each PFT and the heights of the upper and lower layers of the canopy of each PFT. The percentage of each PFT is based on the plant block in the grid. The sum of the percentages of all of the PFTs is 100%. The proportions of lakes, wetlands, glaciers and cities refer to their proportions in the grid.

**Table 1:** Surface data needed for the CLM.

Surface data	Resolution	Ensemble method
Glaciers(percentage)	0.5°	Area averaged values
Lakes(percentage)	1°	Area averaged values
Wetlands(percentage)	1°	Area averaged values
Mud and sand(percentage) Clay (percentage)	5 min	Distribution of soil in the extension range of the largest area in the grid cell
Soil colors	2.8°	Classification of soil colors in the extension range of the largest area in the grid
PFTs(percent of vegetated land)	0.5°	Area averaged values (a maximum of four full PFTs can be selected)
Stem and leaf area indexes (monthly)	0.5°	Area averaged values
Canopy heights (upper and lower layers)	0.5°	Area averaged values

### Data Preparation

In addition to the description of the physical processes of the land surface, the land-surface model is sensitive to the atmospheric driving field. Therefore, the quality of the atmospheric driving field is an important factor to the models simulations^44^. In other words, the spatial-temporal resolution of atmospheric driving field and observation data determines the performance of the land-surface model. The atmospheric driving field of the CLDAS integrates data from various sources such as ground observation, satellite observation and numerical simulation products using integration and assimilation techniques. This product includes temperature, pressure, specific humidity, wind speed, precipitation and solar shortwave radiation. The atmospheric driving field of the CLDAS has a spatial resolution of 1/16° × 1/16° and a temporal resolution of 1 h, and this meets the study requirements for spatiotemporal precision of input data.

**Figure 8:**
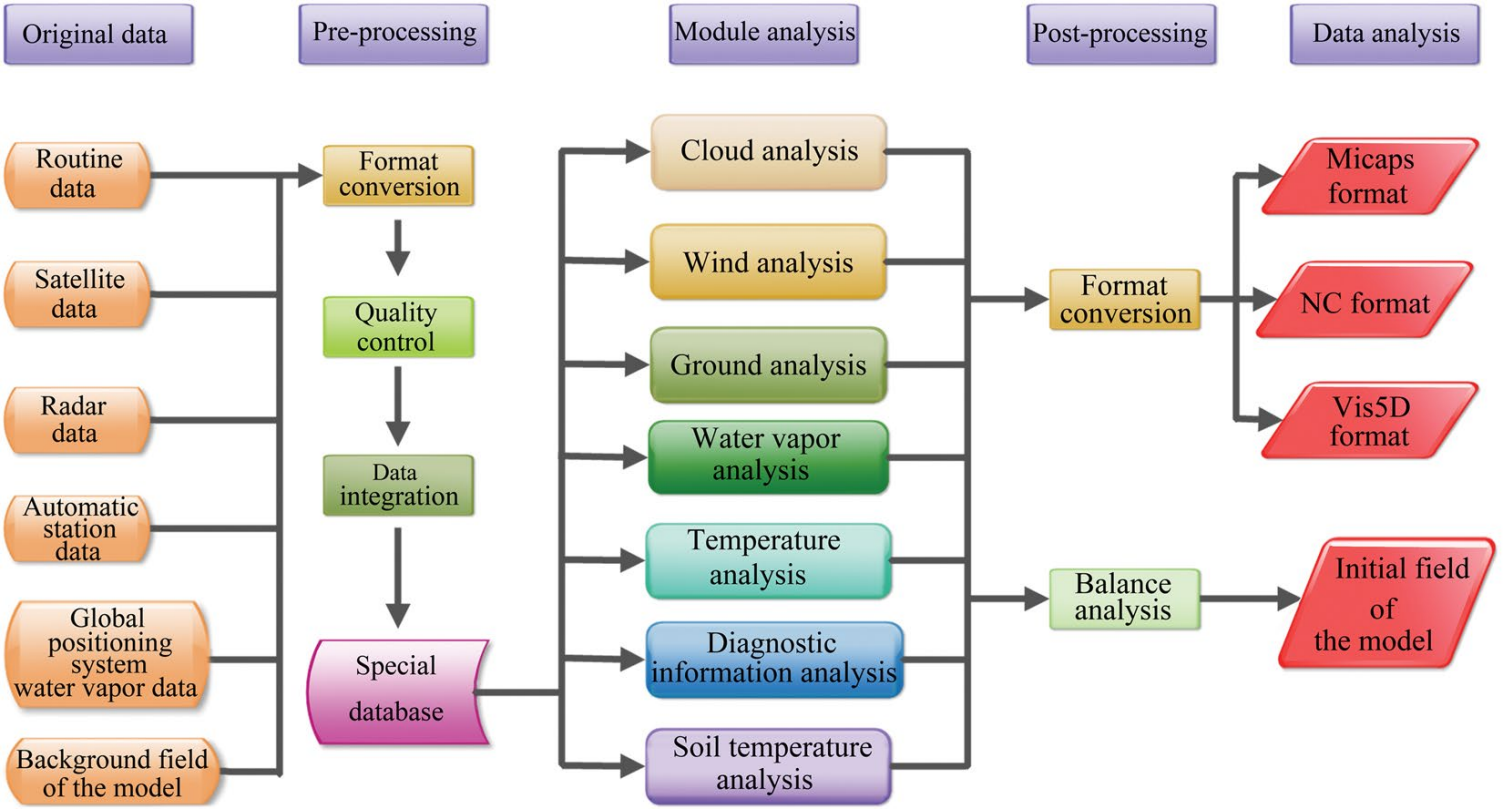
Flowchart of the Local Analysis and Prediction System (LAPS).

The integration of the temperature, pressure, humidity and wind speed data in the CLDAS is implemented using the LAPS/Space and Time Mesoscale Analysis System (STMAS). The LAPS is a comprehensive analysis system for multi-source data, including five major function modules—wind analysis, ground analysis, temperature analysis, cloud analysis and water vapor analysis. The analyses must be carried out according to the order shown in Fig. 8, and the diagnostic analysis can be carried out on the analysis results of the five modules to obtain some diagnostic information about the weather. In addition, the numerical model can be accessed via balance analysis and thus, the model’s hot start can be realized.

The STMAS^45^ is a new-generation integration system that was developed within the framework of the LAPS. The variational method for the order of multiple grids is used as the algorithm of the STMAS, which is relatively different from the conventional LAPS. In terms of its function, the STMAS replaces the ground analysis of the LAPS with the ground analysis module and the wind analysis and temperature analysis of the LAPS with the STMAS3D module, respectively. The input, output, the cloud analysis, water vapor analysis and balance analysis of the STMAS continue to rely on the LAPS. The developers of the STMAS plan to gradually assimilate the diabatic initialization technology in the LAPS to create an independent system.

The multi-grid method was initially used to solve differential equations. A relatively coarse grid can allow a relatively low-frequency oscillation mode to converge rapidly. The multi-grid method was later introduced to data assimilation. The use of the objective function of a coarse grid in analyzing an error longwave can result in the rapid correction of the longwave of the analysis field. Afterwards, the use of the objective function of a fine grid in analyzing an error shortwave can reduce the confusion among different scales and improve the analysis results. The wavelength determines the correlation scale between lattice points. Therefore, the multi-grid method contains a built-in correlation. An analytical wave does not need a definite covariance. Thus, we can control the correlation scale of variation by controlling the number of lattice points. We can adjust the range of influence of the observed information by altering the coarseness of the grid to adjust the error covariance matrix of the background field. Under circumstances in which the accurate covariance is unknown, matrix B can be simplified into a diagonal matrix. The diagonal elements are the error variances of the background. Thus, the demand for computation resources is reduced. In the variational assimilation technique for the order of the multi-grid method, the objective function on each grid is expressed in the following form:

5 $${J}^{({\rm{n}})}=\frac{1}{2}{X}^{(n)T}{X}^{(n)}+\frac{1}{2}{({H}^{(n)}{X}^{(n)}-{Y}^{(n)})}^{T}{O}^{(n)-1}({H}^{(n)}{X}^{(n)}-{Y}^{(n)})\,(n=1,2,3\ldots ,N)$$

where $Y={Y}_{obs}-H{X}_{b}$ and $X={X}_{a}-{X}_{b}$, $O$ represents the observation error covariance matrix, ${X}_{b}$ represents the vector of the background field (forecast field), ${X}_{a}$ represents the vector of the analysis field, ${Y}_{obs}$ represents the constant vector of observation, $H$ represents the bilinear interpolation operator from the model grid to the observation point, $X$ represents the correction vector corresponding to the vector of the model field, which is calculated from the variational data assimilation system, $Y$ represents the interpolation of the observation field and the model field, n represents the n^th^ grid, and $N$ represents the number of grids.

6 $$\{\begin{array}{c}{Y}^{(1)}={Y}^{obs}-{H}^{(1)}{X}^{b}\quad \quad \quad \quad (n=1)\\ {Y}^{(n)}={Y}^{(n-1)}-{H}^{(n-1)}{X}^{(n-1)}\quad \quad (n=2,3,\cdots ,N)\end{array}$$

The STMAS first analyzes the coarsest grid among the multiple grids. When $n=1$, ${Y}^{(1)}$ represents the difference between the projection of the background field of observation on the observation location and the projection of the background field of the model on the observation location, and ${X}^{(n-1)}$ represents the solution or approximate solution of ${J}^{(n-1)}$.

After solving ${J}^{(n-1)}$, ${X}^{(n-1)}$ is interpolated to the finer grid in the n^th^ layer. ${X}^{(n)}$ can be solved through the minimization of ${J}^{(n)}$.

By altering the coarseness of the grid and analyzing the observation data in order from the longwave to the shortwave, we realized the extraction of the observation information of various scales. In this continuous multi-scale analysis process, the observation error covariance matrix of all of the scales (${O}^{(n)}$) is the same as that of the complete observation data because ${O}^{(n)}$ reflects the observation error covariance and is unrelated to the multiple grid layers.

The final analysis result is the superposition of the analysis results of each grid:

7 $${X}^{a}={X}^{b}+{X}_{L}={X}^{b}+\sum _{n=1}^{N}{X}^{(n)}$$

Therefore, unlike the three-dimensional variational (3D-Var) method, the multi-grid method does not confuse the longwave information with the shortwave information in the observation data. Using the 3D-Var method will bring about a relatively larger error in the analysis. This problem is more prominent when analyzing China’s Xinjiang region, which has extremely unevenly distributed observation data. In contrast, the application of the multi-grid method to the preparation of the CLDAS data effectively assures the accuracy of this study’s input data.

The main sources of the hourly CLDAS precipitation grid data include the integrated regional hourly precipitation produced by the National Meteorological Information Center of China, the hourly precipitation retrieved from the Fengyun-2E geostationary satellite by the National Satellite Meteorological Center of China^46^ and the Climate Prediction Center Morphing Technique (CMORPH) integrated satellite precipitation produced by the Climate Prediction Center of the US National Oceanic and Atmospheric Administration. The ground-observed precipitation data used in this study is from the hourly precipitation observed at more than 30,000 automatic observation stations in China. Quality control (the climatological threshold value check, the regional threshold value check, the temporal consistency check and the spatial consistency check) is carried out on the precipitation data collected at more than 30,000 automatic observation stations (including both national and regional automatic observation stations) that have been built in China^47^. The CMORPH integrates the precipitation products retrieved from the microwave sensors of multiple satellites and uses infrared cold-cloud data for time extrapolation to obtain a global half-hourly precipitation product with a resolution of 8 km^48^. Shen *et al*.^49^ evaluated this global half-hourly precipitation product, finding that the advantages of ground observation and the retrieval of precipitation from satellite data are effectively integrated in the CMORPH precipitation product. This CMORPH precipitation product is more reasonable in terms of the amount of precipitation and spatial distribution.

The discrete ordinate method proposed by Stamnes *et al*.^50^ is used as the retrieval algorithm for the ground incident solar radiation of the CLDAS data for radiation transfer calculation. The discrete ordinate method can calculate the radiance in any arbitrary direction. Therefore, the discrete ordinate method considers the anisotropy of the solar radiation reflected by the top of the atmosphere. That is to say, the radiance of the solar radiation reflected by the top of the atmosphere in the satellite’s observation direction is first calculated and then converted to the bidirectional visible albedo that is observed by the visible channel of the satellite. During the transmission process of the solar radiation incident from the top of the atmosphere to passing through the atmosphere to reaching the ground, there is a series of physical processes, including interactions with the atmosphere and ground. The following factors are primarily considered in the retrieval model: (1) absorption by the ozone layer, (2) multiple molecular Rayleigh scattering, (3) multiple scattering and absorption by cloud droplets, (4) absorption by water vapor, (5) multiple scattering and absorption by aerosols and (6) multiple reflection by the ground and atmosphere^32,51^.

This study uses the high spatiotemporal resolution land-surface atmospheric driving data of the CLDAS produced by the National Meteorological Information Center of China Meteorological Administration (temporal resolution: 1 h; spatial resolution: 1/16° × 1/16° and approximately 6.25 km; time scale: 2009–2012; elements: atmospheric temperature, pressure, specific humidity, wind speed, precipitation and shortwave solar radiation) to drive the CLM3.5 to conduct a numerical simulation of the land surface. The process is repeated 10 times to spin up the CLM model. A basically stable initial field of the model is generated to obtain a high temporal-spatial resolution soil temperature dataset. In addition, the bilinear interpolation method is used to interpolate the soil temperature grid data of the CLDAS to the observation stations (Fig. 6) to verify and analyze the hourly data generated by the CLDAS model and the matched sample data generated by observation. In this way, we are able to understand the distribution of soil temperature in the Xinjiang region and the changes in the soil temperature of each layer.

The CLM3.5 can simulate the temperatures of the soil of 10 layers. The depths of the node layers are 0.007 m, 0.0279 m, 0.0623 m, 0.1188 m, 0.212 m, 0.366 m, 0.619 m, 1.038 m, 1.727 m and 2.846 m, respectively. Comprehensively considering the observation depths of the national soil temperature stations of China and the typical temperatures of the soil at various depths, this study selects three soil layers, namely, layer 1 (at a depth of 5 cm), layer 2 (at a depth of 20 cm) and layer 3 (at a depth of 80 cm), which are observed at 105 soil temperature observation stations in Xinjiang (the stations’ locations are shown in Fig. 6), for soil-temperature verification experimentation.

### Validation method for the simulation results

The data are verified in this study to objectively analyze the accuracy of the spatial prediction method. First, the bilinear interpolation method is used to interpolate the planar raster data for the soil temperature simulated using the model to the 105 observation stations in Xinjiang to compare the model simulated temperatures with the observations. We select the root-mean-square error (*RMSE*), the mean absolute error (*MAE*), the mean error (*ME*) and the correlation coefficient (*COR*) for the aforementioned validation (Table 2).

**Table 2:** Four statistical indicators for examining the simulation precision of the model.

Names of the statistical indicators	Equations
*RMSE*	$RMSE=\sqrt{\frac{{\sum _{i=1}^{n}({V}_{oi}-{V}_{pi})}^{2}}{n}}$
*MAE*	$MAE=\frac{1}{n}\cdot \sum _{i=1}^{n}ABS({V}_{oi}-{V}_{pi})$
*ME*	$ME=\frac{1}{n}\cdot \sum _{i=1}^{n}({V}_{oi}-{V}_{pi})$
*COR*	$COR={\rm{corr}}({V}_{oi},{V}_{pi})=\frac{\mathrm{cov}({V}_{oi,}{V}_{pi})}{{\sigma }_{{V}_{oi}}{\sigma }_{{V}_{pi}}}$

*V*_*oi*_: observed soil temperature value (K); *V*_*pi*_: simulated soil temperature value (K); n: number of stations.
